# Heterogeneity and proliferation of invasive cancer subclones in game theory models of the Warburg effect

**DOI:** 10.1111/cpr.12169

**Published:** 2015-02-03

**Authors:** M. Archetti

**Affiliations:** ^1^ School of Biological Sciences University of East Anglia Norwich NR4 7TJ UK

## Abstract

**Objectives:**

The Warburg effect, a switch from aerobic energy production to anaerobic glycolysis, promotes tumour proliferation and motility by inducing acidification of the tumour microenvironment. Therapies that reduce acidity could impair tumour growth and invasiveness. I analysed the dynamics of cell proliferation and of resistance to therapies that target acidity, in a population of cells, under the Warburg effect.

**Materials and methods:**

The dynamics of mutant cells with increased glycolysis and motility has been assessed in a multi‐player game with collective interactions in the framework of evolutionary game theory. Perturbations of the level of acidity in the microenvironment have been used to simulate the effect of therapies that target glycolysis.

**Results:**

The non‐linear effects of glycolysis induce frequency‐dependent clonal selection leading to coexistence of glycolytic and non‐glycolytic cells within a tumour. Mutants with increased motility can invade such a polymorphic population and spread within the tumour. While reducing acidity may produce a sudden reduction in tumour cell proliferation, frequency‐dependent selection enables it to adapt to the new conditions and can enable the tumour to restore its original levels of growth and invasiveness.

**Conclusions:**

The acidity produced by glycolysis acts as a non‐linear public good that leads to coexistence of cells with high and low glycolysis within the tumour. Such a heterogeneous population can easily adapt to changes in acidity. Therapies that target acidity can only be effective in the long term if the cost of glycolysis is high, that is, under non‐limiting oxygen concentrations. Their efficacy, therefore, is reduced when combined with therapies that impair angiogenesis.

## Introduction

### The Warburg effect

Changes in intra‐tumoural metabolism are so common in tumour development that they are considered one of the hallmarks of cancer 1. In particular, the switch from aerobic energy production through oxidative phosphorylation to anaerobic energy production through glycolysis [the ‘Warburg effect’ 2] is so common that it is the basis of fluoro‐deoxy‐d‐glucose positron emission tomography (FdG PET) 3, 4, 5, 6, 7. The up‐regulation of glycolysis is associated with poor prognosis and high malignancy, and has implications for therapies 8, 9, 10, 11, 12. For example, as it enables cancer cells to grow under limiting oxygen concentrations, glycolysis counteracts the effect of anti‐angiogenic therapies 13.

As it occurs even under normal oxygen concentrations, however, the Warburg effect cannot be just an adaptation to hypoxia, and it has been suggested that it is, instead, a way for tumour cells to increase their rate of proliferation compared to normal cells, and improve their invasiveness 14, 15, 16, 17, 18 by increasing acidity of the microenvironment. This ‘acid‐mediated tumour invasion hypothesis’ is reasonable as glycolysis induces microenvironment acidification 19, 20, 21, and because an acidic microenvironment promotes the death of normal cells 22, 23, 24, 25, 26, facilitates tumour invasiveness by increasing extra‐cellular matrix degradation 27, inhibits immune reactions 28 and stimulates release of growth factors 29. Mathematical models describing these effects 30, 31, 32, 33 suggest that the hypothesis is plausible and that targeting acidity could help impair tumour progression and malignancy 17, 34, 35, 36, 37, 38.

If the Warburg effect is an adaptation to increase acidity of the microenvironment, however, a new conceptual problem arises. Switching to anaerobic energy production through glycolysis is costly as glycolysis is less efficient than aerobic energy production through oxidative phosphorylation, thus cells must increase glucose flux to maintain sufficient ATP production. If glycolysis increases without concurrent reduction of aerobic metabolism, on the other hand, cells produce their maximum energetic potential and there may be no cost for increased glycolysis. Similarly, glycolysis can have no cost for a cell during episodes of hypoxia. In these cases, the Warburg effect presents no difficulty. A collective action problem arises when increasing glycolysis is costly for a cell, but induces a beneficial effect for the tumour as a whole. A mutant cancer cell with aerobic metabolism in a population of cells with increased glycolysis would not pay that cost and could still exploit the benefits of living in an acidic microenvironment (created by its neighbouring cells). Explaining what prevents this from happening is necessary to justify the acid‐mediated tumour invasion hypothesis, an issue that is addressed by game‐theory models.

### Game‐theory models of the Warburg effect

Acidification of the microenvironment is the result of the diffusion of metabolic products of glycolysis, such as lactic acid and hydrogen ions, in the extracellular space 19, 20. A cell's fitness, therefore, depends on the amount of glycolysis in its neighbourhood, that is, on how many of its neighbouring cells undergo anaerobic metabolism. The diffusible products of glycolysis (differently from ATP, which is a private good for the cell) are, in the language of game theory, public goods, because their effect is not limited to the producer cell. A cell with aerobic metabolism can enjoy a private benefit (efficient energy production) by exploiting a public good (acidity) produced by neighbouring cells with glycolytic metabolism, raising a collective action problem. The appropriate analytical tool to analyse such collective action problems is game theory. While game theory is used in economics to analyse rational behaviour in human decision‐making, evolutionary game theory 39 does not assume rational decisions; mutations that program a cell to adopt a given phenotype determine that cell's strategy, and competition within the populations (in the case of cancer, clonal selection within the body) results in differential fitness (proliferation rates) for different phenotypes. Game theory models of cancer have been developed to study competition between cells 40, 41, 42, tumour–stroma interactions 43 and the dynamics of growth factor production 44, 45, 46.

Basanta *et al*. 47, 48 developed game‐theory models of the Warburg effect in the context of glioma progression, and found that an invasive phenotype is more likely to evolve after the appearance of glycolysis, and that conditions favouring anaerobic glycolysis also favour tumour invasion. One limitation of these models is that they assume that interactions occur between *pairs* of cells. As the products of glycolysis act as diffusible public goods, however, glycolysis should be modelled as a multi‐player public goods game, rather than as a game with pairwise interactions; it is known that games with pairwise interactions do not generally have the same results as multi‐player, collective action (public goods) games, in particular, they underestimate the fitness of populations at mixed equilibria, which often occur in public goods games 49. Few attempts have been made to analyse collective interactions in cancer research 42, 44, 45, 46. Assuming multi‐player interactions is also essential in order to study the effect of therapies that target acidity, as in multi‐player games a cell's fitness is a function of the number of cells with glycolytic metabolism in its neighbourhood; perturbations of this function allow to study the dynamics of therapies. A recent multi‐player public goods game model of the Warburg effect 50, on the other hand, does not take into account invasive cell types, which is a central feature of the ‘acid‐mediated tumour invasion hypothesis’, and does not analyse the long‐term effects of therapies that target acidity.

### Rationale of the analysis

The purpose of this investigation was to analyse the dynamics of a population of cancer cells that can switch to and from anaerobic metabolism and can develop increased motility. A multi‐player public goods game in the framework of evolutionary game theory allows modelling the impact of therapies that target acidity of the microenvironment, by introducing perturbations at equilibrium. Evolutionary game theory can reveal frequency‐dependent effects that are not apparent from other modelling approaches, and frequency dependence can lead to non‐intuitive results. As we shall see, one of these results, the coexistence of different cell types, has fundamental implications for the stability of therapies that target acidity.

## The model

The model is a four‐strategy, multi‐player public goods game, in which the fitness of a strategy depends on collective interactions of a number of cells. Cells can generate energy either through glycolysis (GLY phenotype) or through oxidative phosphorylation (OXI phenotype). Two additional phenotypes with corresponding metabolism, INV‐GLY and INV‐OXI, have increased motility. GLY and INV‐GLY cells pay a cost *c *> 0, due to increased glycolysis. The benefit *b*(*j*) of environmental acidity (induced by glycolysis) accrues to all cancer types, irrespective of whether they have glycolytic metabolism, and is a function of the sum *j* of GLY or INV‐GLY cells in the neighbourhood. The size of the neighbourhood is defined by group size *n* (which depends on diffusion range of products of the glycolysis). The benefit function is modelled by a sigmoid function, which allows description of various types of synergistic effects 50, 51, 52. To take into account the possibility that high levels of glycolysis are detrimental to tumour cells (self‐poisoning), we use a double sigmoid function, monotonically increasing for *j *< *d* and monotonically decreasing for *j *> *d*:

$$b\left(j\right)=\left\{\begin{array}{c} b_{1}\left(j\right)j\leq d\cdot \\ b_{2}\left(j\right)j>d\cdot \end{array}\right.$$

where

$$b_{1}\left(j\right)=\left[l_{1}\left(j\right)-l_{2}\left(0\right)\right]/\left[l_{1}\left(d\cdot n\right)-l_{1}\left(0\right)\right]$$

$$b_{2}\left(j\right)=1-\left[l_{2}\left(j,y\right)-l_{2}\left(d\cdot n,y\right)\right]/\left[l_{2}\left(n,1\right)-l_{2}\left(d\cdot n,1\right)\right]$$

are the normalized versions of the logistic functions

$$l_{1}\left(j\right)=\frac{1}{1+e^{s_{1}\cdot \left(h_{1}-\frac{j/n}{d}\right)}}$$

$$l_{2}\left(j,y\right)=\frac{y}{1+e^{s_{2}\cdot \left(h_{2}-\frac{j/n-d}{1-d}\right)}}$$

The parameter *d* describes the value of *j* at which the benefits of acidity are overcome by its deleterious effects; for *j* < *d·n*, the function is monotonically increasing and has an inflection point at *h*_1_ and steepness *s*_1_; for *j* > *d·n*, the function is monotonically decreasing and has an inflection point at *h*_2_ and steepness *s*_2_ (with 0 < *h*_1_,*h*_2_ ≤* *1 and *s*_1_,*s*_2_ > 0); the additional parameter *y* measures the maximum damage of self poisoning.

**Figure 1:**
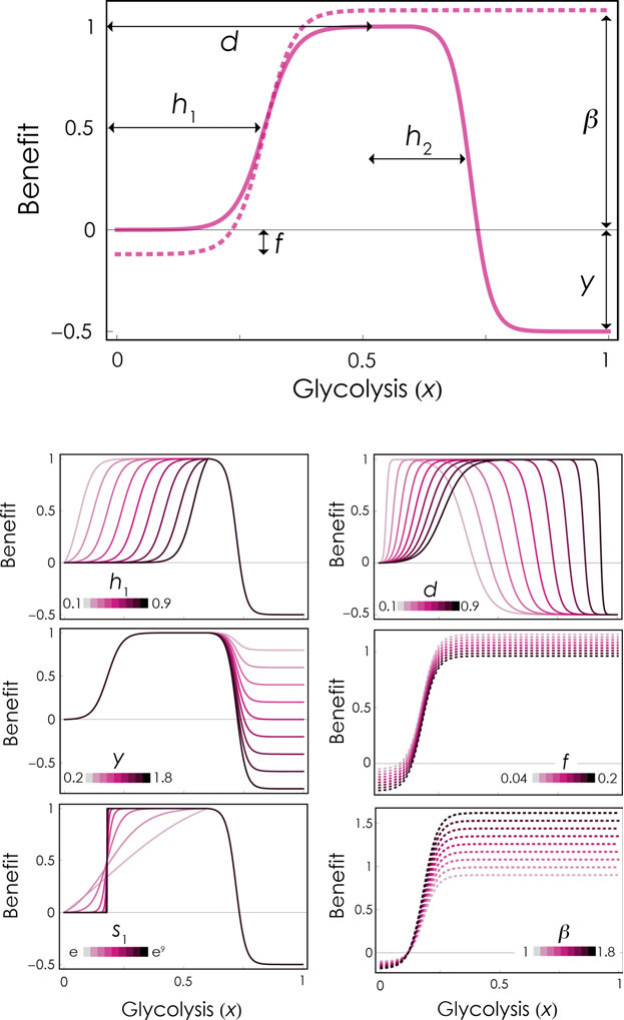
**Effect of the parameters on the benefit function.** The benefit of glycolysis‐induced acidity is plotted as a function of the fraction (*x*) of GLY and INV‐GLY cells. The continuous line shows the benefit *b*(*j*) for the non‐invasive phenotypes GLY and OXI; the dashed line shows the benefit β[*b*_1_(*j*) − *f*] for the invasive phenotypes INV‐GLY and INV‐OXI. Self‐poisoning occurs for *x *> *d* for the non‐invasive phenotypes. The inflection point of the function is *h*_1_ for *x *<* d* and *h*_*2*_ for *x *> *d*. The steepness of the function is *s*_1_ for *x *<* d* and *s*_*2*_ for *x *> *d*. The maximum amount of self‐poisoning is *y*. The maximum cost of motility is *f*. The benefit of motility is β. The larger plot shows the functions for GLY and for INV‐GLY for *h* = 0.5, *d* = 0.6, *s* = 20, *y* = 1.5, *f* = 0.1, β = 1.2. The smaller plots show the effect of changing individual parameters. In all cases, group size *n* = 20; *s*_*2*_ = 20; *h*_*2*_ = 0.5.

In a large population with no assortment, one can approximate the analysis by assuming an infinite, well‐mixed population, and the fitnesses of GLY and OXI cells are given by respectively

$$W_{GLY}\left(x\right)=\sum_{j=0}^{n-1}\left(\begin{array}{c} n-1\\ j \end{array}\right)x^{j}{\left(1-x\right)}^{n-1-j}\cdot b\left(j+1\right)-c$$

$$W_{OXI}\left(x\right)=\sum_{j=0}^{n-1}\left(\begin{array}{c} n-1\\ j \end{array}\right)x^{j}{\left(1-x\right)}^{n-1-j}\cdot b\left(j\right)$$

where 0 ≤ *x *≤ 1 is the fraction of GLY and INV‐GLY cells in the population. We assume that invasive phenotypes, because of their ability to move away when acidity is high, do not suffer self‐poisoning. The fitnesses for the invasive phenotypes are, therefore:

$$W_{INV-GLY}\left(x\right)=\sum_{j=0}^{n-1}\left(\begin{array}{c} n-1\\ j \end{array}\right)x^{j}{\left(1-x\right)}^{n-1-j}\cdot \beta \left[b_{1}\left(j+1\right)-f\right]-c$$

$$W_{INV-OXI}\left(x\right)=\sum_{j=0}^{n-1}\left(\begin{array}{c} n-1\\ j \end{array}\right)x^{j}{\left(1-x\right)}^{n-1-j}\cdot \beta \left[b_{1}\left(j\right)-f\right]$$

where the additional parameter β measures the maximum benefit achieved by the cells and *f* measures the cost of increased motility. The fitness functions therefore assume that the invasive phenotype pays a net cost for motility at low values of *x*, whereas when acidity (*x*) is high enough, the benefit of motility overcomes its cost. Parameters are summarized in Table 1, and their effect on the benefit function is summarized in Fig. 1.

**Table 1:** Summary of the parameters

*c*	Cost of glycolysis
*x*	Frequency of cells with increased glycolysis in the population
*j*	Number of cells with increased glycolysis in the neighbourhood
*n*	Neighbourhood size
*d*	Value of (*j*/*n*) above which *b* decreases with *j* (self‐poisoning)
*h*_1_	Inflection point of *b* for *j *<* d·n*
*h*_2_	Inflection point of *b* for *j *>* d·n*
*s*_1_	Steepness of *b* at *h*_1_
*s*_2_	Steepness of *b* at *h*_2_
β	Benefit of completely acidic environment
*y*	Cost of maximum level of self poisoning
*f*	Cost of increased motility
*ξ*	Fraction of the original acidity left after therapy

While in models with two strategies or pairwise interaction, an analytical characterization of the dynamics and of the equilibria is often possible, complexity of the present model is beyond analytical tractability. Thus, I analysed the dynamics by simulation assuming standard, deterministic replicator dynamics. Therapies that reduce acidity of the tumour microenvironment are modelled as perturbations of the equilibrium: when cell type frequencies are stable, the contribution of each GLY or INV‐GLY cell to acidity is reduced to a fraction *ξ* of the original contribution. The population is then allowed to evolve under the new conditions, in which each cell with a glycolytic metabolism only contributes *ξ* to the overall acidity of the microenvironment.

## Results

### Coexistence or extinction

When the invasive phenotype produces energy by anaerobic metabolism (INV‐GLY), it generally goes to fixation, driving both GLY and OXI phenotypes to extinction; an invasive phenotype that produces energy by oxidative phosphorylation (INV‐OXI), instead, generally coexists with the GLY phenotype (while the OXI phenotype becomes extinct). Tumour fitness is higher in the former case. The final composition of the population does not depend on the initial frequencies of the three phenotypes (Fig. S1).

**Figure 2:**
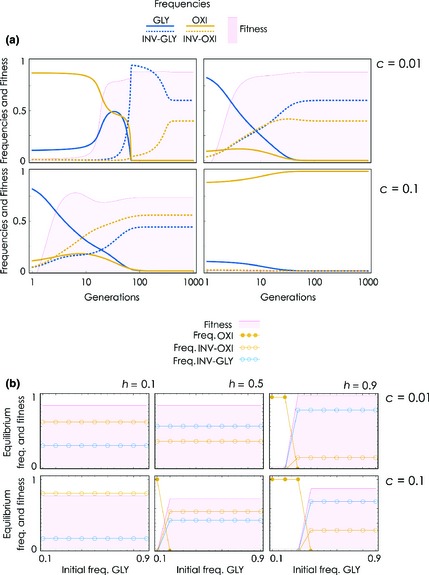
**Effect of initial frequencies on the dynamics with both types of invasive cell.** (a) Changes over time of the frequencies of the four cell types and of tumour fitness starting from different initial frequencies. *h*_1_ = *h*_2_ = 0.5, *d* = 0.6, *s*_1_ = *s*_2_ = 20, *y* = 1.5, *f* = 0.1, β = 1.2, *n* = 20. (b) Frequencies and tumour fitness at equilibrium as functions of the initial frequency of the GLY type; the initial frequency of the other cell types is 0.01 for INV‐GLY and INV‐OXI, and all the rest are OXY cells.

If both invasive phenotypes are present, however, they (INV‐GLY or INV‐OXI) always coexist at equilibrium, unless the initial frequency of the GLY phenotype is low enough and *c* is high enough (Fig. 2). In this latter case, there are not enough GLY cells for glycolysis to evolve and therefore not enough acidity for invasive phenotypes to proliferate. In what follows, unless stated otherwise, I assume that aerobic metabolism (OXI) is the original condition of the cell population and a small fraction (0.001, unless stated otherwise) of GLY and invasive cells (INV‐GLY or INV‐OXI) arise in this population.

### Effect of the parameters on the dynamics

If the only invasive phenotype in the population has aerobic metabolism (INV‐OXI) (Fig. S2), it will coexist with GLY if *h*_1_ is low; if *h*_1_ is high, and if *d* or *c* are high enough, instead, the OXI phenotype can even drive INV‐OXI and GLY cells to extinction. When two types coexist, fitness decreases with *h*_1_ and *d*, with *c* and β, and increases with *s*_1_; when the INV‐GLY phenotype goes to fixation, however, fitness is higher for higher values of *h*_1_; GLY and INV‐OXI can coexist and frequency of GLY increases with *d* and decreases with *c*,* s*_1_ and β; however, if *h*_1_ is high enough and *d* or *c* is too high, both GLY and INV‐OXI become extinct and are replaced by OXI (Fig. S2). In short: a mutant INV‐OXI phenotype generally leads to a polymorphic population in which it coexists with the GLY type or (if the cost of glycolysis is too high or the benefits of acidity require a large faction of cells with glycolytic metabolism) to a monomorphic population made of OXI cells.

If only the invasive phenotype with glycolytic metabolism (INV‐GLY) is present (Fig. S2), INV‐GLY either becomes extinct (for high *d* and high *h*_1_), leading to the fixation of the OXI phenotype, or drives the other types to extinction (if *c* is low); it can only coexist with OXI if *c* is high, whereas GLY and OXI coexist only if the cost of motility is extremely high compared to its benefit (high *f*, low β) (Fig. S2). In short: a mutant INV‐GLY phenotype generally leads to a monomorphic population, either of itself or (if the cost of glycolysis is too high or benefits of acidity require a large faction of cells with glycolytic metabolism) of the OXY type.

**Figure 3:**
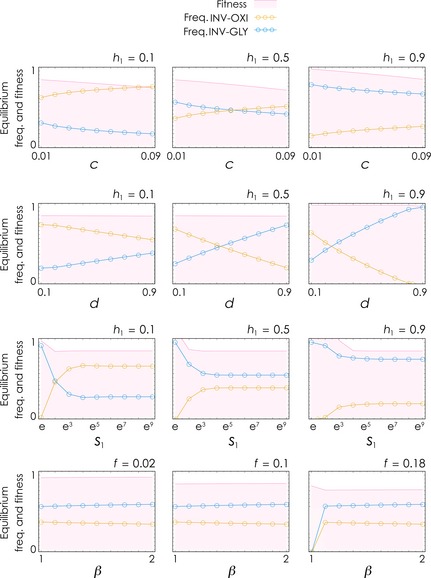
**Effect of the parameters on the equilibrium and on tumour fitness with both types of invasive cell.** Tumour fitness and cell type frequencies as a function of *c* (the cost of glycolysis for GLY and INV‐GLY cells), *d* (the fraction of GLY and INV‐GLY cells above which self‐poisoning starts) or *s* (the steepness of the benefit function at the inflection point for *x *< *d*), for different values of *h*_1_ (the inflection point of the benefit function for *x *< *d*, or as a function of β (the scaling factor of the benefit for invasive cells) for different values of *f* (the cost of motility). Other parameters: *d* = 0.6, *c* = 0.01, *h*_2_ = 0.5, *n* = 20, *s*_1_ = *s*_2_ = 20, *y* = 1.5, *f* = 0.1; β = 1.2.

While the two invasive mutant types individually lead to either a monomorphic population of OXY cells or to a stable coexistence of GLY and INV‐OXI, when both invasive phenotype are present, the dynamics are more complex, but the results are rather simple: OXI and GLY types become extinct, unless the cost of motility is large enough (*f *> 0.2) (Fig. 3). When the two invasive phenotypes coexist, the frequency of INV‐GLY decreases with *c* (the cost of glycolysis) and increases with *h*_1_ (that is, INV‐GLY is more common when higher acidity is necessary for proliferation and motility), *d* (that is, INV‐GLY is more common when self‐poisoning is triggered by higher acidity) and β (that is, INV‐GLY is more common when the benefit of motility is higher).

In all cases, the details of self‐poisoning (values of *y, h*_2_ and *s*_*2*_) are largely irrelevant to the dynamics (even in a model in which self‐poisoning is introduced in the invasive types).

### Effects of therapies that reduce acidity of the microenvironment

The short‐term effect of reducing acidity is a reduction in tumour fitness. If the population is at a polymorphic equilibrium, however, the frequencies of the two types change following the new acidity level: the fraction of GLY cells increases, leading to a new increase in acidity and tumour growth; if the reduction in acidity is small (high *ξ*), the population has, at the new equilibrium, approximately the same fitness as before treatment (Fig. S3), although invasiveness declines. In the presence of the INV‐GLY phenotype, instead, tumour growth declines and remains constant, but invasiveness does not decline as the population is, in this case, monomorphic.

**Figure 4:**
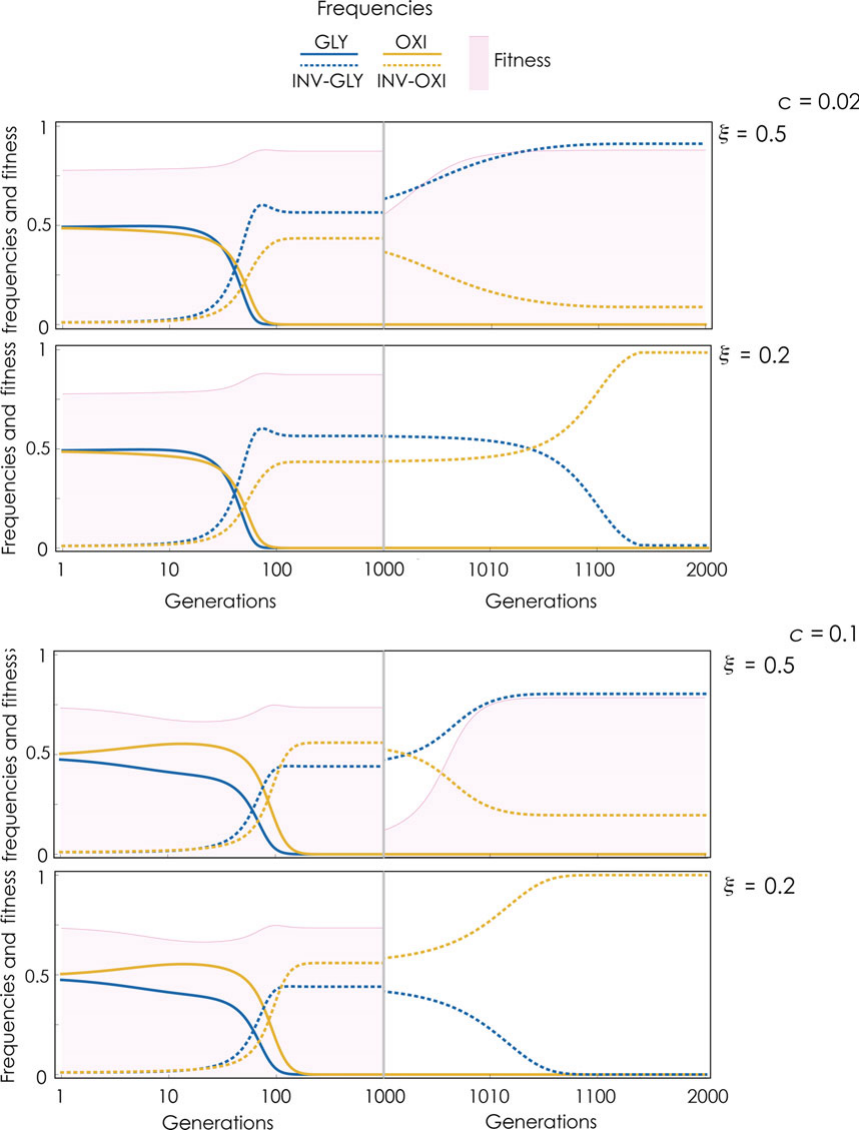
**Dynamics of therapies that reduce acidity with both invasive cell types.** Acidity is reduced to a fraction *ξ* = 0.5 or 0.2 at generation 1000. *h*_1_ = 0.5, *d* = 0.6, *h*_2_ = 0.5, *n* = 20, *s*_1_ = *s*_2_ = 20, *y* = 1.5, *f* = 0.1; β = 1.2.

**Figure 5:**
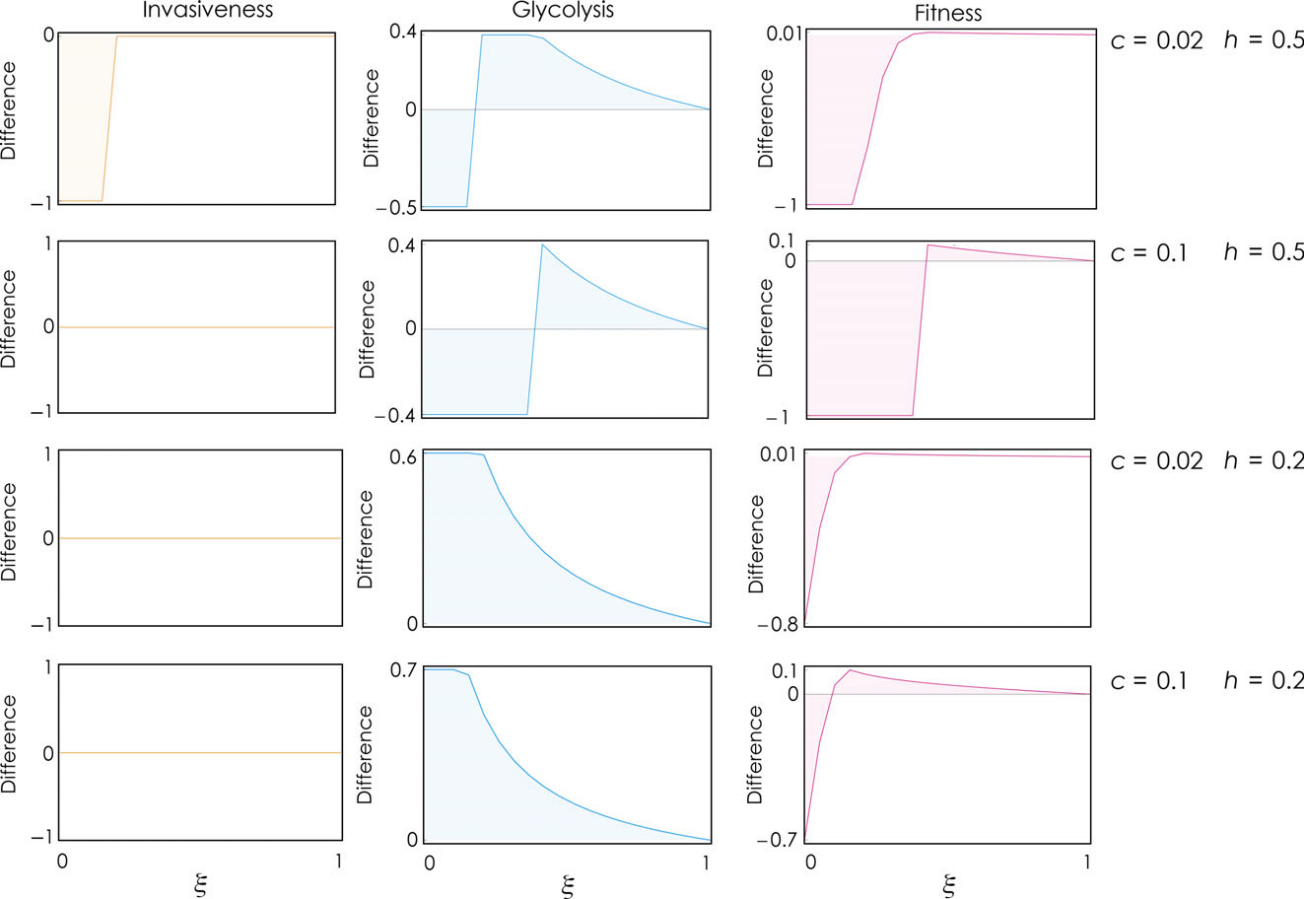
**Summary of the effect of therapies that reduce acidity.** After the population has reached an equilibrium, acidity is reduced to a fraction *ξ* of the original value, and the population reaches a new equilibrium. The plots show the difference in fitness, glycolysis (*x,* the sum of the frequencies of the GLY and INV‐GLY phenotypes) and invasiveness (the sum of the frequencies of the INV‐GLY and INV‐OXI phenotypes) between the new and the original equilibrium, as a function of *ξ,* for different values of *c* and *h*. Other parameters: *d* = 0.6, *h*_2_ = 0.5, *n* = 20, *s*_1_ = *s*_2_ = 20, *y* = 1.5, *f* = 0.1; β = 1.2.

If both invasive phenotypes are present (Fig. 4), reducing acidity has the same immediate effect: a reduction in tumour cell proliferation. If acidity is not reduced enough, however (*ξ* is high) the fraction of INV‐GLY cells increases at the expense of INV‐OXI, irrespective of the cost of glycolysis, and a similar fitness level is restored at the new equilibrium; in fact, fitness at the new equilibrium can be even higher (Fig. 5). Invasiveness remains unaffected as both types present at the initial equilibrium have high motility. If the reduction of acidity is large enough (*ξ* is high), the treatment can effectively stop tumour cell proliferation; the frequencies evolve to a stable monomorphic population of either the INV‐GLY phenotype (if the cost of glycolysis is low, for example, under low oxygen concentrations, when glycolysis is more efficient), or the INV‐OXI phenotype (if the cost if high). Invasiveness, however, is not reduced overall.

Finally, a lower value of *ξ*, that is, a larger reduction in acidity, is necessary to make the treatment effective when the value of *h*_1_ is low (Fig. 5), that is, when few cells are enough to induce enough acidity in the microenvironment to enable cells with high motility to proliferate.

## Discussion

### Summary of the results

As acidity induced by glycolysis enhances proliferation of cancer cells irrespective of their metabolism, selection for increased glycolysis within a tumour is frequency‐dependent and leads to a stable coexistence of cells with aerobic and anaerobic metabolism. Such a polymorphic population can be invaded by mutant subclones with increased motility, and is the basis for the evolution of resistance to therapies that target acidity. Analysing the dynamics and stability of the system reveals how such therapies could be more effective. These points will be discussed in detail below.

### The Warburg effect leads to intra‐tumour heterogeneity

A game theoretic model of tumour proliferation has been analysed, in which cells can adopt aerobic or anaerobic metabolism, and can mutate to have high motility. High motility has a cost for the cell, but it also has an advantage if the microenvironment is acidic enough. Gatenby and others 15, 16, 17, 18 have proposed that the benefit conferred by acidity, rather than aerobic energy production *per se*, is the adaptive value that promotes the Warburg effect in tumours – a hypothesis that seems justified by the fact that glycolysis is often up‐regulated, even under normal oxygen concentrations.

This ‘acid‐mediated tumour invasion hypothesis’, however, raises a conceptual problem; as the effects of acidity are not restricted to cells with glycolytic metabolism, glycolysis is a public good, that is, a form of cooperation among cancer cells. A mutant cell that reverted to aerobic energy production would benefit from more efficient metabolism while still enjoying the advantage of an acidic microenvironment produced by its neighbouring cells. Game theory can explain how glycolysis is maintained in spite of this ability of mutant cells to ‘free‐ride’ on glycolysis of other cells.

As we have seen, if the rate of proliferation of a cancer cell is a non‐linear function of acidity produced by glycolysis, frequency‐dependent selection leads to coexistence of glycolytic and non‐glycolytic cell types within the tumour. Coexistence results from the fact that products of glycolysis act as non‐linear public goods, and therefore enable coexistence of cells with aerobic and anaerobic metabolism. As we have seen here, this form of cooperation between cancer cells remains stable in the presence of mutant cells with increased motility. These invasive cells require an acidic microenvironment to thrive and would not be able to proliferate in the absence of enough glycolytic cells. The Warburg effect, therefore, leads to heterogeneity for energy production within the tumour, which enables meastasis of invasive phenotypes, which can also persist as a stable polymorphism.

### Heterogeneity can lead to resistance against therapies that target acidity

The stable polymorphism predicted by the model is the basis of the development of resistance to therapies that aim to reduce acidity of the microenvironment. Impairing glycolysis may induce short‐term reduction of tumour growth and invasiveness, and it has been suggested 17 that this may be achieved through glucose deprivation, inhibition of the glycolytic pathway, of glucose transport, or by targeting (for example, by inhibiting its transcription) the hypoxia‐inducible factor HIF. It appears that existing therapies that aim to inhibit glycolysis alone do not show significant anti‐tumour effects, and are generally used in combination with other therapies 17.

The predicted polymorphism of a cell population under the Warburg effect may help explain the evolution of resistance to therapies that target acidity. While a monomorphic population can adapt to new conditions (hence, therapies) only by clonal selection of new mutants that arise in the course of the treatment, if a population is polymorphic it can adapt to new conditions simply by changing the frequencies of the cell types already present. This seems to be the case here. Unfortunately, therefore not only is the Warburg effect itself an adaptation of the tumour to improve proliferation and invasiveness; in addition, the resulting heterogeneity is an obstacle for therapies that target acidity.

### Increasing oxygen level can make therapies more effective

As we have seen, therapies that reduce acidity can be effective if the cost of glycolysis is high enough. When glycolysis provides higher benefits to cells, that is under limiting oxygen concentrations, the relative cost of glycolysis is lower; hypoxia, therefore, would promote the evolution of resistance to therapies that target acidity. On the other hand, allowing a sufficient oxygen level would increase the relative cost of glycolysis, reducing the possibility of relapse. This means that effectiveness of such therapies would be compromised by concurrent administration of drugs that impair angiogenesis.

This seems to be in contrast with the conclusion of Basanta *et al*. 48 that anti‐angiogenic treatments would be beneficial for therapies that target glycolysis [see also 17] as they reduce the fraction of glycolytic (invasive or not) cell types. The discrepancy, however, may be due only to a difference in the modelling approach. In Basanta *et al*. 48, angiogenesis was induced by growth factors produced by glycolytic cell types, and therapies that reduced angiogenesis had a direct impact on these two types, which are indeed the ones whose growth was reduced (in their model) by anti‐angiogenic treatment. In the model presented here, instead, cancer cells do not induce angiogenesis themselves. Basanta *et al*. 48 showed that the fraction of glycolytic invasive cell type declined with the cost of glycolysis, which is consistent with the predictions of the present model.

We have also seen that therapies that target acidity must be more effective to be stable in the long term, as the number of glycolytic cells required to produce acidity decreases. An empirical measure of this number, of the diffusion range of products of glycolysis and, in general, of the other parameters of the model would help establish whether the proposed therapies 17 that target acidity could be evolutionarily stable.

### Experimental tests

While the main results (frequency‐dependent selection leading to stable heterogeneity and resilience to changes in acidity) are largely independent of their exact values, it would be useful to measure the parameters of the model. Estimating the shape of the benefit function requires measuring the diffusion range of the products of glycolysis (which determines neighbourhood size); if this was known, it would be possible to estimate the shape of the benefit function by measuring the growth of populations with different fractions of glycolytic cells. The cost of glycolysis should also be measured.

While aerobic metabolism is less efficient than aerobic energy production through oxidative phosphorylation, and while we have assumed a cost for increased glycolysis, there are cases in which increasing glycolysis or switching to anaerobic metabolism is not costly. Cells can increase glycolysis without reducing their aerobic metabolism, thus creating their maximum energetic potential, which may be helpful (and not costly) during periods of fast growth with non‐limiting resources; glycolysis can also be cost‐free during episodes of hypoxia. In these cases, clearly the Warburg effect poses no difficulty, and frequency‐dependent selection is not necessary to explain the up‐regulation of glycolysis. Frequency‐dependent selection, driven by collective interactions, explains however how the Warburg effect makes sense when glycolysis has a cost.

If there is a cost for glycolysis, a mixed equilibrium is stable as the marginal advantage of increasing glycolysis declines with the fraction of cells with high glycolysis, and at some point it is offset by the cost, even without self‐poisoning 50. In other words, when glycolysis is costly for a cell and produces a collective benefit for the tumour, a mixed equilibrium is stable even without self‐poisoning. On the other hand, if there is no cost for glycolysis, the glycolytic type would go to fixation in the absence of self‐poisoning. With self‐poisoning, however, the fraction of glycolytic cells remains at intermediate levels simply because too much glycolysis is deleterious, irrespective of frequency‐dependent selection.

### Future theoretical work

As we have seen here, in models that allow both types of cells (glycolytic or not) to give rise to mutants with increased motility, types with higher motility always invade a polymorphic population, and therefore spread within the tumour (provided the cost of motility is low compared to its benefits). Motility, therefore, is not an essential component of the model and could be ignored in future analyses. This does not mean, of course, that increased motility is not an essential feature of tumour dynamics; it simply means that, because we can always expect increased motility to be favoured by clonal selection, there is no need to model it explicitly. This was not obvious in the light of previous results 47, 48, 50. With hindsight, not taking motility explicitly into account is an advantage for future studies, as models with only two types are more amenable to analytical study.

The most significant omission in this and previous models of the Warburg effect is the lack of explicit spatial structure. The model assumes a well‐mixed population, and spatial structure is known to have significant effects on the dynamics of cooperative interactions 52. An extension of this multi‐player game to a spatially structured population would be an important theoretical improvement. Furthermore, the Warburg effect often characterizes only the hypoxic domain of the tumour, and the tumour uses lactate from glycolysis as a source of energy through a lactate shuttle between hypoxic and oxygenated cells 53, 54, 55, 56. This spatial effect has been recently modelled 56, assuming however pairwise interactions between groups of cells, a model that would also benefit from an extension to multi‐player interaction.

## Conclusions

Overall, the results presented here support the idea that glycolysis‐induced acidity is an adaptation of the tumour to improve competition against healthy cells and invasiveness, although they also raise issues with the idea that acidity can be exploited as a treatment, and make predictions about interactions with anti‐angiogenic therapies that appear to contradict previous results. The long‐term effects of therapies based on perturbations of microenvironment acidity are rather complex; analysing cell interactions in the framework of evolutionary game theory can, besides helping understand the logic of the Warburg effect, help understand the dynamics of such therapies.

## Supporting information


**Fig. S1.** Effect of initial frequencies on the dynamics with only one type of invasive cell.

**Fig. S2.** Effect of the parameters on the equilibrium and on tumour fitness with only one type of invasive cell.

**Fig. S3.** Dynamics of therapies that reduce acidity with only one invasive cell type.
