# Supplementary figures and images for: Heterogeneity and proliferation of invasive cancer subclones in game theory models of the Warburg effect
Source: Cell Prolif. 2015 Feb 3;48(2):259–69. doi: 10.1111/cpr.12169 (PMC4964921; doi:10.1111/cpr.12169)

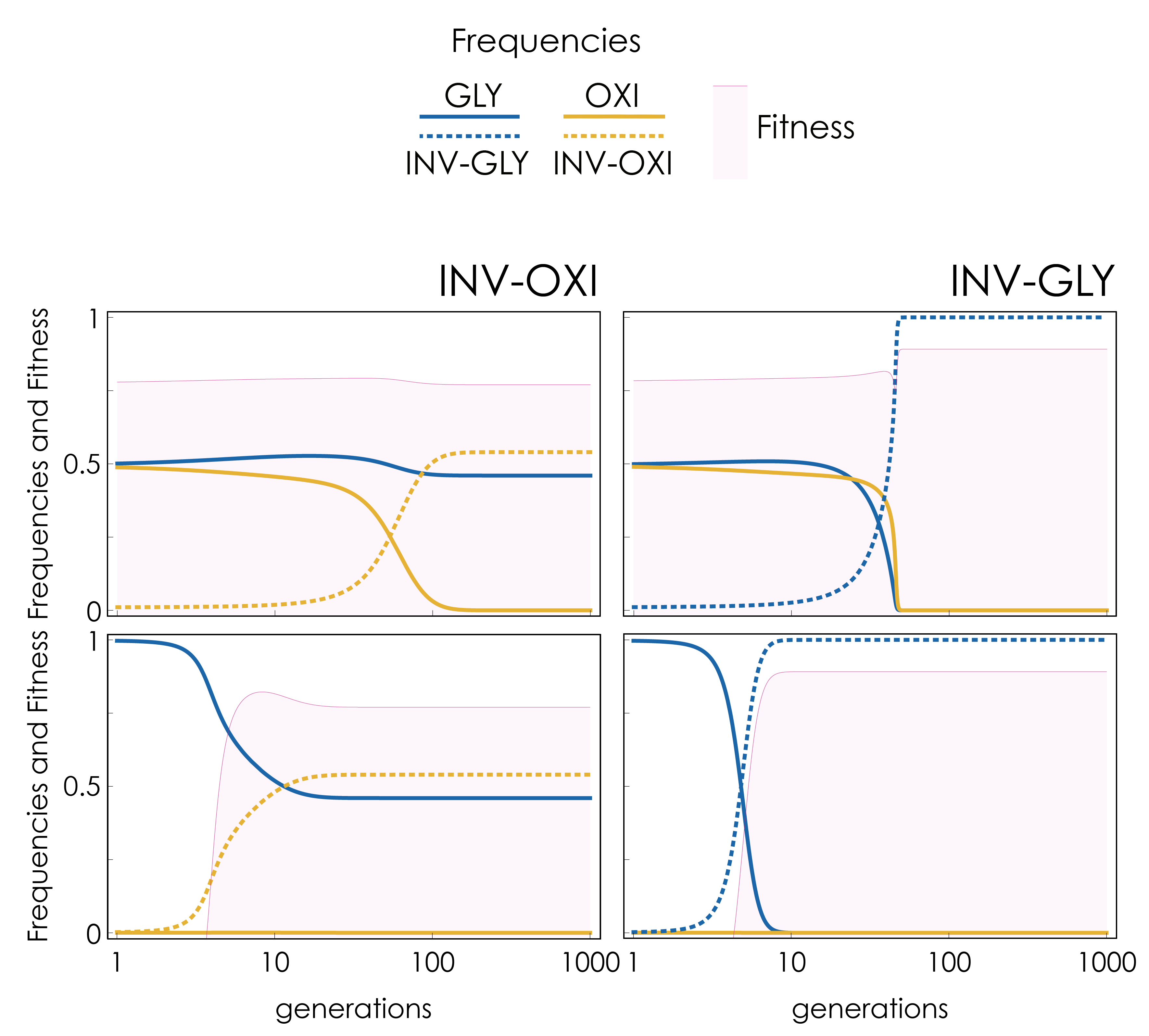

Supplement: Supplementary file 1 — Fig. S1. Effect of initial frequencies on the dynamics with only one type of invasive cell. [file CPR-48-259-s001.tif]

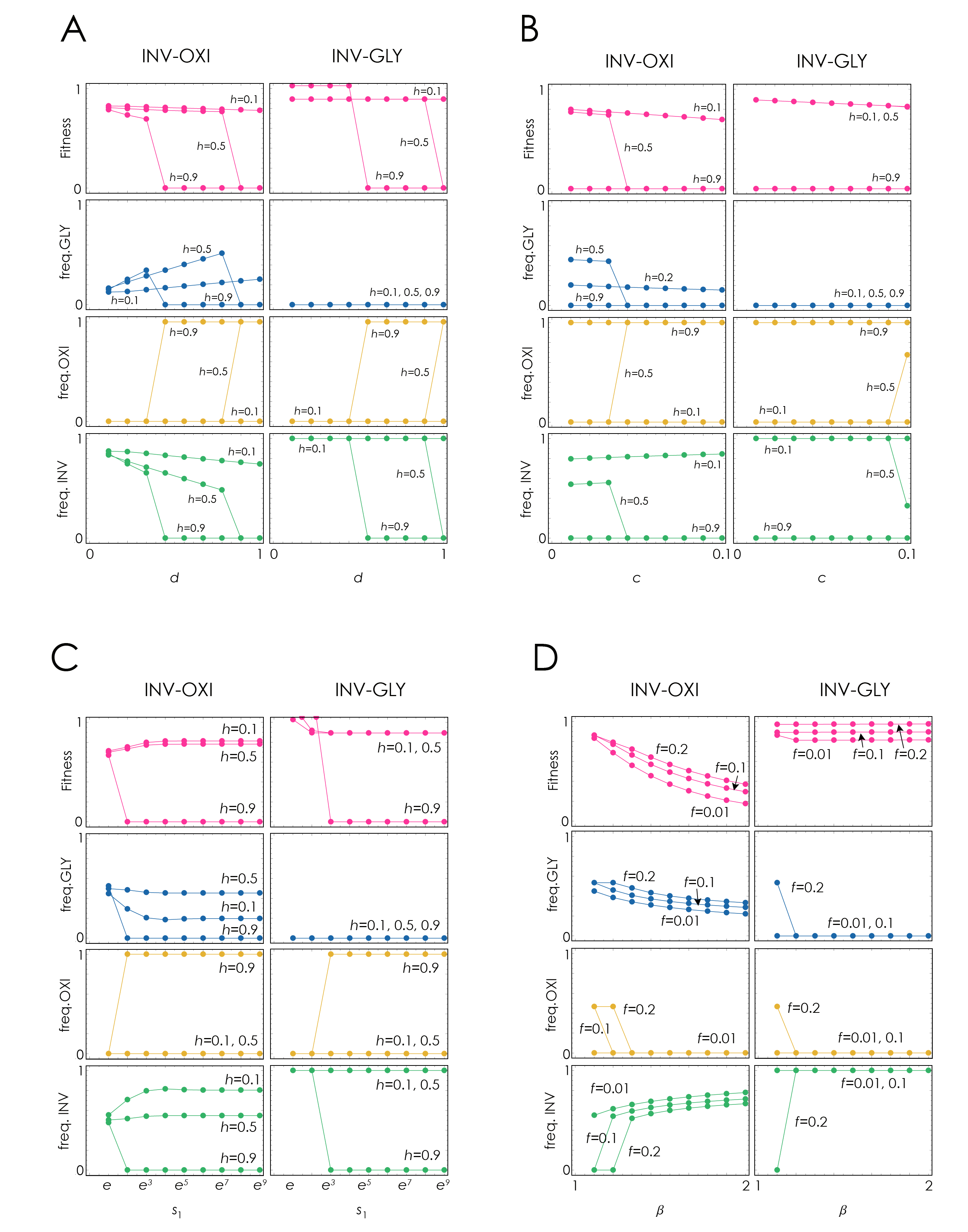

Supplement: Supplementary file 2 — Fig. S2. Effect of the parameters on the equilibrium and on tumour fitness with only one type of invasive cell. [file CPR-48-259-s002.tif]

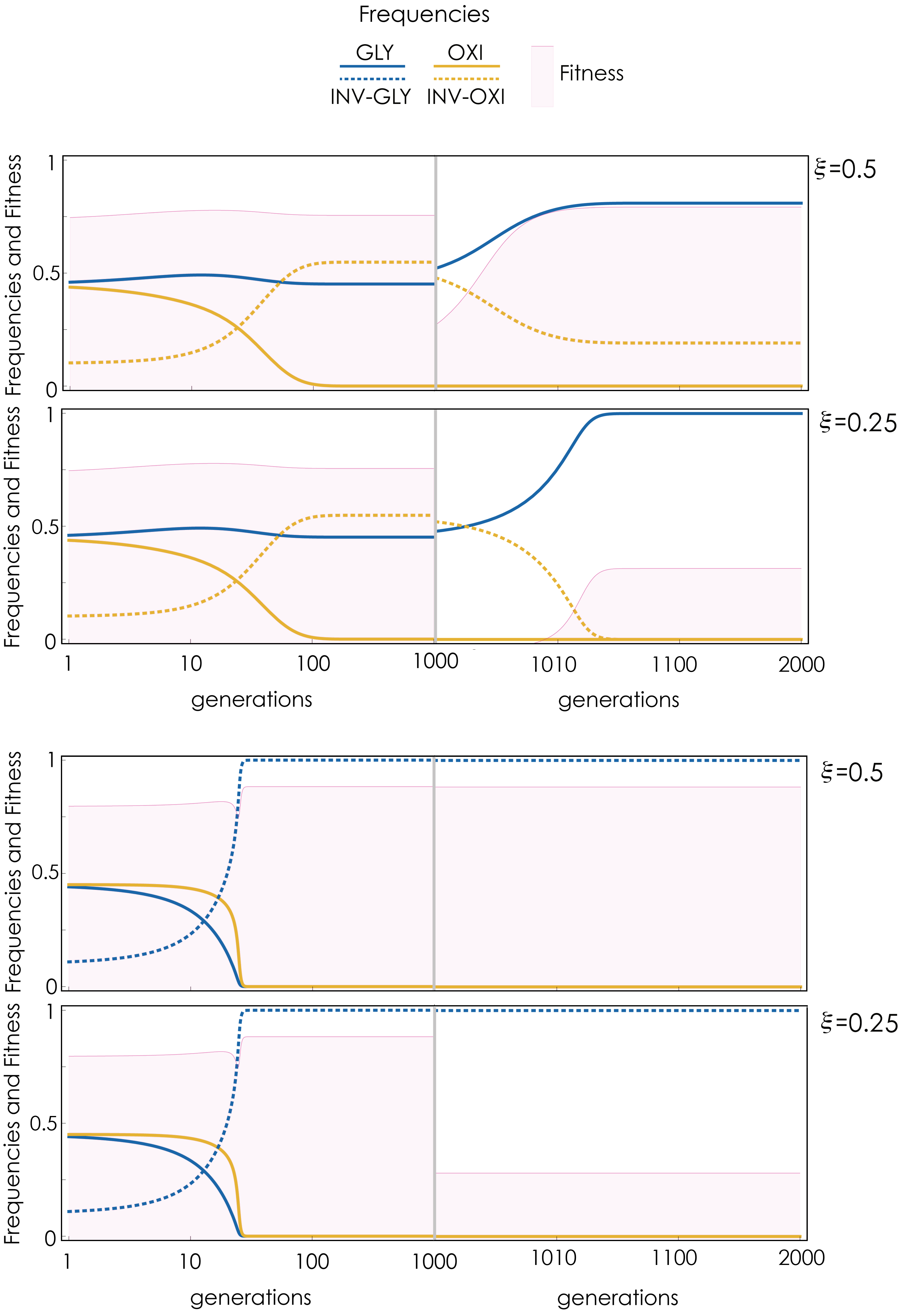

Supplement: Supplementary file 3 — Fig. S3. Dynamics of therapies that reduce acidity with only one invasive cell type. [file CPR-48-259-s003.tif]
